# Carbon and Nitrogen Sources Influence Parasitic Responsiveness in *Trichoderma atroviride* NI-1

**DOI:** 10.3390/jof10100671

**Published:** 2024-09-26

**Authors:** Víctor Javier García-Sánchez, Karina Lizbeth Sánchez-López, Juana Jazmín Esquivel Méndez, Daniel Sánchez-Hernández, José Antonio Cervantes-Chávez, Fidel Landeros-Jaime, Artemio Mendoza-Mendoza, Julio Cesar Vega-Arreguín, Edgardo Ulises Esquivel-Naranjo

**Affiliations:** 1 Unit for Basic and Applied Microbiology, Faculty of Natural Sciences, Autonomous University of Queretaro, Queretaro 76140, Mexico; vgarcia10@alumnos.uaq.mx (V.J.G.-S.); karinalsanchezlopez@gmail.com (K.L.S.-L.); jesquivel05@alumnos.uaq.mx (J.J.E.M.); daniel.sanchez@posgrado.ecologia.edu.mx (D.S.-H.); jose.antonio.cervantes@uaq.mx (J.A.C.-C.); landeros@uaq.mx (F.L.-J.); 2 Faculty of Agriculture and Life Sciences, Lincoln University, Lincoln 7647, New Zealand; artemio.mendoza@lincoln.ac.nz; 3 Laboratory of AgroGenomic Sciences, National School of Higher Studies, National Autonomous University of Mexico, Guanajuato 37684, Mexico; jvega@enes.unam.mx

**Keywords:** biocontrol, parasitism, host sensing, catabolic repression, genes related to mycoparasitism, hydrolytic enzymes

## Abstract

Parasitic species of *Trichoderma* use hydrolytic enzymes to destroy the host cell wall. Preferent carbon and nitrogen sources suppress the expression of genes related to parasitism. Here, different nutrients were evaluated in the parasitic isolated NI-1, which was identified as *Trichoderma atroviride*. The genes *cbh1* and *chb2* (cellobiohydrolases), *bgl3.1* (endoglucanase), and *pra1* and *prb1* (proteinases) were poorly expressed during the interaction between NI-1 and *Phytophthora capsici* on PDA. However, gene expression improved on minimal medium with preferent and alternative carbon sources. Dextrin and glucose stimulated higher transcript levels than cellulose, sucrose, and glycerol. Also, ammonium stimulated a stronger parasitic responsiveness than the alternative nitrogen sources. During interaction against different phytopathogens, NI-1 detects their host differentially from a distance due to the *cbh1* and *cbh2* genes being only induced by *P. capsici*. The *pra1* and *ech42* genes were induced before contact with *Botrytis cinerea* and *Rhizoctonia solani*, while when confronted with *P. capsici* they were stimulated until contact and overgrowth. The *prb1* and *bgl3.1* genes were induced before contact against the three-host assayed. Overall, *T. atroviride* prefers to parasitize and has the capacity to distinguish between an oomycete and a fungus, but nutrient quality regulates its parasitic responsiveness.

## 1. Introduction

Mycoparasitism is a process in which a fungus alters its hyphal growth and begins the biogenesis of specialized structures to attack, penetrate, and consume its fungal host [1]. Mycoparasitism of many species of the genus *Trichoderma* has been known for over 80 years [2,3], and is a trait exploited to protect and improve crops. However, it has largely remained unknown how *Trichoderma* recognizes its host and which players are key in regulating the genes that encode enzymes involved in destroying and consuming the host cell wall or are involved in the biogenesis of specialized structures [1].

*Trichoderma* species have been widely recognized as a successful biocontrol agent to antagonize many agriculturally important oomycetes and fungal plant pathogens by different mechanisms such as direct parasitism, antibiosis, and competition. Furthermore, they promote plant growth and induce plant-resistance [1,4]. During parasitism, *Trichoderma* produces hydrolytic enzymes that destroy the main structural components of the host cell wall such as proteases (*pra1* and *prb1* genes), chitinases (*ech42* and *chit* genes), and glucanases (*bgl3.1*), with the main aim of obtaining nutrients from the host. However, competition for nutrients and space could be another reason. The parasitism process begins with chemotropic growth towards the host mycelia; once in-touch recognition proceeds, coiling around the host hypha and formation of hooks like appressorium to penetrate the hyphae and reach the cytoplasm, then the parasite breaks down host cellular components, lysing the host cell and causing subsequent death of the phytopathogen [5]. It has been proposed that *Trichoderma* cells constitutively secrete a low concentration of cell wall degrading enzymes, which are involved in delivering diffusible molecules from the host cell wall, and act as signals to detect the host and induce genes related to mycoparasitism (GRMs) [5,6,7], but the inducers have not yet been identified [1]. Furthermore, parasitic activity varies among *Trichoderma* species or different isolates and the potential host [3], suggesting that different strategies are used to parasitize a host. *T. harzianum* ThHP-3 triggers a host-specific response, regulating the differential expression of genes encoding hydrolytic enzymes and the production of secondary metabolites and transporters during interaction with *Fusarium* or *Colletotrichum* as hosts [8]. Also, the expression levels of some GRMs were different between *T. virens* and *T. atroviride* when confronted against *Rhizoctonia solani* AG5 and AG2, suggesting a different molecular mechanism among parasites to detect and respond to a host [9]. Furthermore, the *lam1.3* gene encoding a β-1,3-glucanase was upregulated when *T. harzianum* confronted *Sclerotinia sclerotium*, but not in *T. hamatum* [10], suggesting some genetic variation to recognize or attack a host, although these differences could also be associated with different approaches or quantity/quality of nutrients.

Some genes’ GRMs encode hydrolytic enzymes that *Trichoderma* utilizes to consume alternative carbon or nitrogen sources. In vitro, these genes are derepressed by nutrient starvation, induced by the specific substrate and/or fungal cell wall, and repressed by preferent carbon and nitrogen sources [11,12,13,14,15,16,17], indicating that their expression is under carbon and nitrogen catabolic repression (CCR/NCR) [10]. These molecular mechanisms involve processes focused on economizing energy in fungi, prioritizing the assimilation of preferential carbon or nitrogen sources, and, consequently, repressing genes encoding proteins involved in the degradation and utilization of alternative sources [11,12]. In *Trichoderma*, the presence of glucose activates CCR via the AMPc pathway, which activates protein CRE1 [11] by dephosphorylation, being able to reach the nucleus and represses the expression of genes involved in metabolizing alternative carbon sources such as alcohols, organic acids, and polysaccharides [13]. Regarding nitrogen metabolism, ammonium and glutamine activate NCR to repress genes encoding enzymes that metabolize alternative nitrogen sources, but during starvation, the transcriptional factor AreA is activated to promote the metabolism of alternative nitrogen sources [14,15]. In this regard, *prb1, ech42, bgl3.1,* and *pra1* are derepressed by starvation and induced with fungal cell walls [16,17,18]. Notably, these genes are not detected when preferent carbon and/or nitrogen sources are added in the presence of the host cell wall as inducers [17]. Furthermore, the genes encoding hydrolytic enzymes related to parasitism are triggered by diffusible factors before colonization of a fungal host [16,17,18], but the molecule acting as an inducer has not yet been identified.

Most information is focused on understanding how *Trichoderma* antagonizes the plant pathogens ascomycetes and basidiomycetes [5] using very limiting nutritional conditions. The aim of this work was to determine the effect of different nutrient sources to understand the molecular responsiveness during the parasitic interaction of a native *Trichoderma* strain NI-1 against *P. capsici*, *R. solani,* and *B. cinerea*. Our results highlight a predominant parasitic behavior under conditions where metabolic repression could work and a complex network of molecular mechanisms for sensing what kind of prey is available to parasitize, activating specific combat strategies.

## 2. Materials and Methods

### 2.1. Strains and Culture Conditions

The *Trichoderma atroviride* NI-1 is a native strain isolated from the rhizosphere of *Eriobotrya japonica* in Toliman municipality, Queretaro, Mexico. The oomycete *Phytophthora capsici* D3 strain was originally isolated from a chili cultivar at Dolores-Hidalgo, Guanajuato [19,20]. The fungal plant pathogens *Botritys cinerea* and *Rhizoctonia solani* AG2 were previously documented in confrontation against *Trichoderma virens* [21]. Fungal strains were propagated on PDA medium (DIFCO) and *P. capsici* on V8 juice agar (50 mL of V8 juice–CaCO_3_–agar) and incubated at 28 °C in darkness. *P. capsici* D3 was constantly kept on agar through the tests intercropping PDA-V8-PDA. Since we focused on *T. atroviride* parasitism against an oomycete and two true fungi, parasitism was used instead of mycoparasitism.

### 2.2. NI-1 Strain Identification

To ensure genetic uniformity, monosporic cultures were made from stock internal ceparium. Fresh mycelium from NI-1 growth on PDA medium and conidia was harvested with distilled water. Then, 100 µL of dilution 10^−8^ of spores were inoculated by extension on PDA medium supplemented with chloramphenicol at 50 µg/mL (USB Corporation, Cleveland, OH, USA) and Triton X-100 at 0.5% (Bio basic, Markham, ON, Canada). The isolate was grown for three days at 28 °C under constant light. The colonies obtained were inoculated on fresh PDA medium.

Genomic DNA was extracted from mycelium cultures in a potato dextrose broth (PDB) incubated at stirring at 160 rpm for 72 h at 28 °C. Mycelia were collected by filtration, treated with N_2(l),_ and pulverized. The equivalent of 500 µL of powder mycelia was added to a 1.5 mL plastic tube with 600 µL of urea buffer (urea 8 M, NaCl 0.5 M, Tris 20 mM, EDTA 20 mM, SDS 2%, pH 8) and vortexed for 2 min and incubated at room temperature for 30 min. Phenol–chloroform extraction was performed two times as follows: 600 µL of phenol–chloroform–isoamyl alcohol (50:49:1) was added to the mixture, vortexed for 3 min, centrifugated at 11,000× *g* for 10 min, and the supernatant was transferred to a new 1.5 mL tube. After this, one volume of isopropanol was added, softly homogenized and centrifugated for 11,000× *g* for 10 min. The pellet was washed with 500 µL of 70% ethanol, mixed and centrifugated as previously mentioned, and dried at room temperature. The final pellet was resuspended on 100 µL of TE buffer (Tris 10 mM, EDTA 1 mM, pH 8) and treated with 0.1 UμL^−1^ RNAse I (ThermoFisher Scientific, Waltham, MA, USA) at 37 °C for 30 min. The pellet was stored at −20 °C.

The internal transcribed spacer region and translation elongation factor 1-alpha were amplified using primers for ITS and Tef-1α listed in Table S1. Amplicons were purified using a QIAquick PCR purification kit (QIAGEN, Hilde, Germany) and sequenced at the Laboratorio Nacional de Genómica para la Biodiversidad (LANGEBIO) facilities. 

Concatenated sequences were aligned and manually adjusted when needed in relation to reference sequences using MEGA software (11.0.13). Alignment was analyzed using maximum parsimony (MP), maximum likelihood (ML) and Bayesian inference (BI) by PAUP 4.0a [22], RaxML 7.2.6 [23], and Mr. Bayes 3.2.5 [24] software, respectively. MP analysis was conducted using a heuristic search of 1000 replicates with TBR algorithm for the branches exchange and 1000 bootstrap replicates for branch support (BMP). ML analysis was carried out using the GTR+G substitution model and 1000 bootstrap replicates (BML). BI analysis was performed with the GTR+G+I model under the following parameters: ngen = 1,000,000, samplefreq = 100, and nruns = 4; posteriorly a burn-in of 25% to calculate the posterior probability (PP). We selected *T. pubescens* as the outgroup taxon. NCBI accession numbers used for phylogenetic analysis are listed in Table 1.

### 2.3. Antagonism Assays by Nonvolatile Compounds

To detect antibiosis mediated by diffusible metabolites, 5 mm mycelium plugs of NI-1 were inoculated on the center of PDA plates covered with a cellophane layer and incubated at 27 °C for 48 h. The cellophane layer with *T. atroviride* NI-1 mycelium was then removed, and a 5 mm plug of *P. capsici*, cut from the edge of the young colony, was inoculated at the center and incubated at 27 °C. At the same time, a control assay was carried out with inoculated *P. capsici* on fresh PDA plates, and the diameter of colonies was measured before the control reached the border of the plate, using ImageJ software (1.51j8v, NIH, Bethesda, MD, USA).

### 2.4. Antagonism Assays by Volatile Compounds

For antibiosis mediated by volatile organic compounds (VOCs), *T. atroviride* NI-1 and *P. capsici* mycelium plugs were placed on different PDA plates, placed together on the opposite side, and sealed with a plastic film to form a closed chamber. As a control, *P. capsici* was inoculated on both PDA plates, and both were sealed on opposite sides. Cultures were incubated at 27 °C, placing the VOC chamber with *T. atroviride* NI-1 growing at the bottom, and *P. capsici* growing at the top. When the control nearly reached the border of the PDA plates, the colony diameter was photographed and measured by ImageJ software (1.51j8v, NIH, Bethesda, MD, USA).

### 2.5. Parasitism Assays

The parasitic activity was assessed using a dual culture comprising mycelium plugs of *T. atroviride* NI-1 and *P. capcisi,* which were inoculated on opposite sides of Vogel’s minimal medium (VMM) plates (sucrose 1%, 100× concentrated mineral salts solution 2.5% consisting of KH_2_PO_4_ 2.5%, NH_4_NO_3_ 1%, MgSO_4_ 0.1%, CaCl_2_ 0.05%, and trace element solution 0.05% ZnSO_4_, 0.01% FeSO_4_, 0.005% CuSO_4_, 0.002% MnSO_4_, 0.002% H_3_BO_3_, and 0.002% Na_2_MoO_4_), at 1.5 cm from the border. The interaction zone was directly observed using an optical microscope (Leica ICC50 E, Leica Microsystems Ltd., Heerbrugg, Schweiz) equipped with a Leica camera to analyze parasitic structures, and images were processed using Leica LAS EZ software (V3.4.0, Leica Microsystems Ltd., Heerbrugg, Schweiz). Dual cultures were carried out on medium plates inoculated on opposite sides 1.0 cm from the border to analyze the overgrowth rate. Overgrowth rates were scored once *Trichoderma* mycelium and phytopathogen colonies came into contact with each other.

### 2.6. Effect of Different Carbon Sources on GRM Expression

To analyze the effect of carbon sources on parasitism, modifications on VMM were made by replacing sucrose with glucose (PQM Fermont, Monterrey, Mexico), dextrin from potato starch (Sigma-Aldrich, Saint Louis, MO, USA), glycerol (J.T. Baker, Phillipsburg, NJ, USA), sucrose, or cellulose (Sigma-Aldrich, Saint Louis, MO, USA) at 1%. Dual cultures were performed; inoculated *T. atroviride* NI-1 plugs and *P. capsici* were placed on opposite sites at 1.5 cm from the border of plates and then inoculated until both colonies contacted each other. Around 3 cm^2^ of mycelium was collected 1 cm after they came into contact, frozen in liquid nitrogen, and used for RNA extraction.

### 2.7. Effect of Different Nitrogen Sources on GRM Expression

To analyze the influence of nitrogen on parasitism, VMM was modified, replacing NH_4_NO_3_ (J.T. Baker, Phillipsburg, NJ, USA) with NaNO_3_ (Meyer, Ciudad de México, Mexico) and L-arginine (Golden-Bell, Ciudad de México, Mexico) at 1%. To prepare a mineral solution concentrate, salts were dissolved and sterilized by filtration using Whatman filters 0.45 µm (Little Chalfont, Buckinghamshire, UK). Interactions were performed with modified minimal medium inoculating *T. atroviride* NI-1 and *P. capsici* D3 mycelium plugs placed at 1.5 cm from the border of the plates. The interaction was compared on PDA media. Around 3 cm^2^ of mycelium was collected 1 cm after they came into contact, frozen in liquid nitrogen, and then used for RNA extraction.

### 2.8. Dual Cultures Assay to Analyze Gene Expression before-, during Contact, and Overgrowth 

Dual cultures on VMM with 1% dextrin were performed to analyze *T. atroviride* NI-1’s interactions with plant pathogens, including an oomycete *P. capsici*, and two fungi *B. cinerea*, and *R. solani*. Mycelia was collected at 2, 1, and 0.5 cm before contact, during contact, and about 2 cm overgrowing (Figure S3). Mycelia was frozen in liquid nitrogen and kept at −70 °C for RNA extraction.

The control was carried out following the same approach, but two *T. atroviride* NI-1 mycelium plugs were placed at opposite sites on the plates to confront each other. All interactions were carried out in the dark, and cultures were visually monitored under a safe red light until the mycelia reached the abovementioned stages. Approximately 3 cm^2^ of *T. atroviride* NI-1 mycelia were collected, placed in a 1.5 mL tube, and frozen with liquid nitrogen. Samples were stored at −80 °C until processing.

### 2.9. RNA Extraction and Manipulation

Mycelia samples were homogenized using a tissue grinder and 1 mL of Trizol reagent (Invitrogen, Carlsbad, CA, USA) in 1.5 mL tubes. Mixtures were vortexed at maximum speed for 8 min at room temperature and centrifuged for 1 min at 13,000× *g* at 4 °C. The supernatant (SN) was recovered and mixed with 1 mL of chloroform (J.T. Baker, Phillipsburg, NJ, USA). The two phases were separated by centrifugation at 13,000× *g* at 4 °C for 10 min (^©^ PrismR Labnet International). From the SN, total RNA was insolubilized with a volume of isopropanol at −20 °C for 12 h, and then it was recovered by centrifugation as indicated above. RNA pellet was washed with 1 mL of chilled 70% ethanol–DEPC to eliminate mineral salts. The pellets were then dried at room temperature, resuspended in 20 µL of nuclease-free water (Zymo-Research, Irvine, CA, USA), and 1 µg of RNA samples were treated with 1 U of DNAse I (Thermo Fisher Scientific, Waltham, MA, USA) at 37 °C for 30 min. 

### 2.10. Complementary DNA Synthesis

Oligonucleotides were designed based on the *T. asperellum* CBS 433.97 strain genome [42] and *T. atroviride* IMI 206040 genome [26], except for the *gpd* gene [43]. The nucleotide sequences of the open reading frames of basic proteinase *prb1* (XM024901871), chitinase *ech42* (XM024910215), cellobiohydrolases *cbh1* (XM024906693) and *cbh2* (XM024910837), endo-β-glucanase *bgn*13.1 (XM024901914), and acid proteinase *pra1* (XM024903604) were used to design the oligonucleotides by hand and properties were obtained using Oligo Calc calculator (http://biotools.nubic.northwestern.edu/OligoCalc.html, accessed on 20 June of 2021) [44] (Table S1).

cDNA was synthesized using 1 µg of total RNA previously treated with DNAse. RNA template and 10 pmoles reverse primers (Table S1) were incubated for 5 min to 65 °C before beginning the reaction by addition of 10 mM dNTPs (Thermo Fisher Scientific, Waltham, MA, USA), buffer, and 0.5 units of RevertAid Reverse Transcriptase (Thermo Fisher Scientific, Waltham, MA, USA) for each sample. Reactions were carried out at 42 °C for 60 min and terminated at 70 °C for 10 min. cDNA was stored at −20 °C until use for end-point PCR. 

### 2.11. End-Point PCR to Detect Transcripts

The DreamTaq DNA Polymerase (Thermo Fisher Scientific, Waltham, MA, USA), 10 mM dNTPs (Thermo Fisher Scientific, Waltham, MA, USA), 0.1 mM forward and reverse oligonucleotides, respectively, and 1 µL of previously obtained cDNA was used as a template. Reactions were carried out according to the following conditions: first step at 95 °C/3 min, 25–45 cycles of 95 °C/30 s, 61 °C/45 s, 72 °C/45 s, and a final extension step of 72 °C/3 min. 

### 2.12. Semiquantitative Expression Analysis

Gene expression was determined based on the selection of a cycle in which PCR products exponentially increased before reaching a maximum signal for each transcript according to the Calcáneo–Henández protocol [45] with few modifications. For this approach, end-point PCR was performed under the abovementioned conditions for the first set of five cycles for each reaction, from 25 to 45 (25, 30, 35, 40, and 45), and the amplicons were analyzed by electrophoresis. 

End-point PCR was repeated, increasing cycle by cycle before the sutured signal, and a cycle was chosen before saturation (Table S1). In both cases, total RNA during the interaction with NI-1–*P. capsici* was used as a template to ensure the detection of GRM analyzed. This approach was used to analyze the gene expression of genes listed in Table S1 during three phases of *Trichoderma* parasitism: before contact, during physical contact, and overgrowing, as mentioned above. Amplicon area signals were quantified using ImageLab software (v 6.1.0, Standard Edition, Bio-Rad Laboratories, Inc., Hercules, CA, USA), and expression was normalized using the *gpd* transcript signal as a template-loaded control.

### 2.13. Statistical Analysis

Rstudio (version 2024.04.2, Posit Software, PBC, Boston, MA, USA) and GraphPad Prism 8 (8.0.1, GraphPad Software Inc., San Diego, CA, USA) were used for statistical analysis and graph generation. The experiments were performed in triplicate, and significant differences between treatments were determined using a value of *p* ≤ 0.05. Two-way ANOVA and all means were compared using Dunnett’s test to detect statistical differences between genetic expressions at different confrontations. A nonparametric test was applied when homoscedasticity was not observed.

## 3. Results

### 3.1. Trichoderma NI-1 Has a High Antagonistic Capacity against P. capsici D3

**Figure 1:**
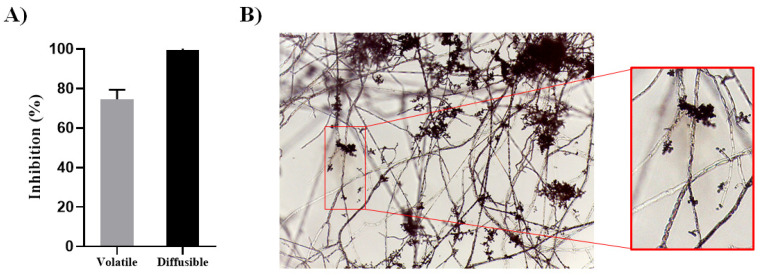
In vitro antagonism against *P. capsici* D3. (**A**) Growth inhibition by diffusible and volatile metabolites. Bars represent the average of at least three treatments. Line upper bars indicate the standard deviation (SD). (**B**) Mycoparasitic behavior of NI-1 against *P. capsici* D3 (10×). Hyaline hyphae of NI-1 coiled around D3 hyphae. Coiling is indicated by the red square.

It has been reported that *P. capsici* D3 is an aggressive plant pathogen [19,20]; therefore, identifying and characterizing biocontrol agents able to antagonize this oomycete opens up major possibilities for establishing better control strategies. The NI-1 strain was isolated from the soil and chosen because of its capacity to antagonize several phytopathogenic fungi. Its antagonistic activity to control *P. capsici* by diffusible and volatile metabolites was analyzed (Figure 1 and Figure S1). Inhibition by diffusible compounds was highly efficient (100%), *P. capsici* was unable to growth and significantly more effective than by volatile organic compounds (VOCs), which was around 70% (Figure 1A). NI-1 was able to colonize the D3 mycelium, developing parasitic structures in the interaction zone in the dual culture test. NI-1 hyphae grew along the hyphae of D3 as a guide, and highly dense coiling structures were observed at some points (Figure 1B). Consistently, these dense coiling structures and overgrowth of the *P. capsici* colony are excellent indicators of parasitic aggressiveness. NI-1 inhibited *P. capsici* D3 growth through multiple mechanisms, suggesting that strain NI-1 could be a suitable biocontrol agent.

### 3.2. NI-1 Strain Is Phylogenetically Related to Trichoderma atroviride 

Two colonies were obtained from monosporic cultures with the same colony characteristics: white and villous (Figure S2A). Both colonies appeared pale green, with a distinctive coconut odor due to 6-pentyl-α-pyrone emitted from the colonies on PDA medium (Figure S2B). In addition, both strains presented a regular concentric colonial morphology with abundant aerial mycelium and rapid radial growth, reaching the border Petri dish (90 mm diameter) after approximately three days. The back of the plate was not pigmented.

**Figure 2:**
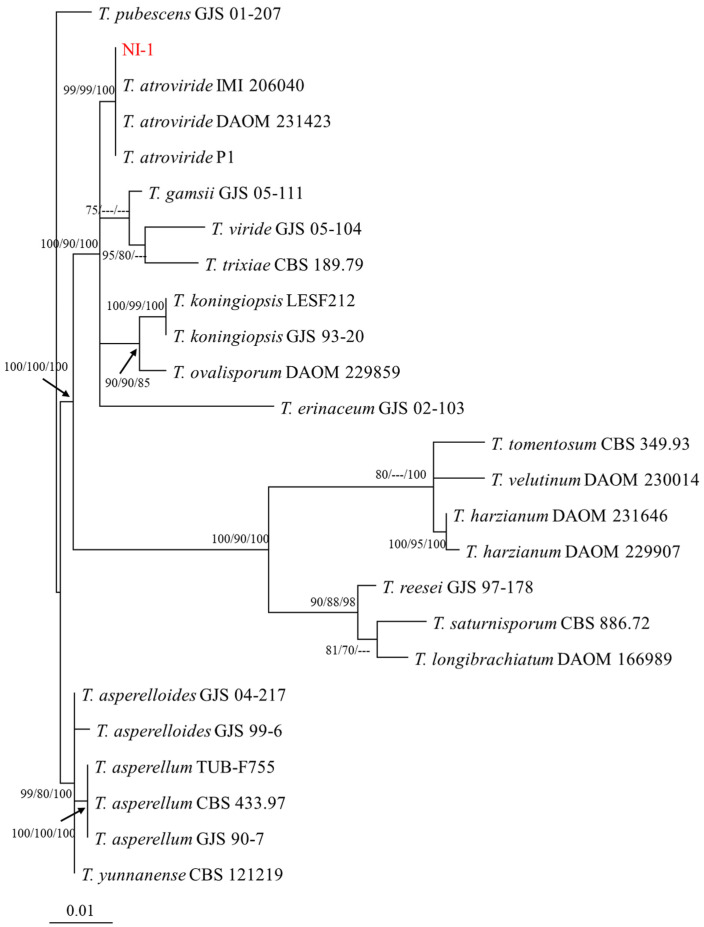
Phylogenetic analysis of NI-1. Phylogram based on the combined ITS and TEF-1α sequences. Support values higher than 70% are shown at the nodes. Values are associated with BMP, BML, and PP, respectively. The accession numbers of the reference sequences are shown in Table 1. NI-1 is shown in red. The scale bar represents nucleotide substitutions per base.

A phylogenetic tree was constructed with 25 ITS and TEF-1α sequences, including our isolate NI-1 and 24 reference sequences obtained from the GenBank repository (Table 1). ITS and Tef-1α alignment between the two isolates showed high similarity, indicating that they were the same strain. The phylogenetic tree and bootstrap values also showed that NI-1 clustered with *Trichoderma atroviride* (Figure 2). In addition to this, we ensured genetic uniformity on the stock spore solution that was subsequently used for the next assays.

### 3.3. Carbon Sources Influence GRM Expression 

**Figure 3:**
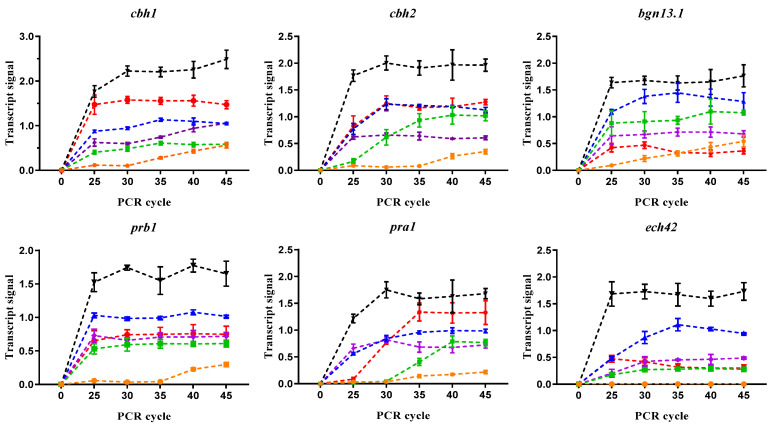
Influence of carbon source on the transcript levels of parasitism-related genes during confrontation with NI-1 and D3. Dual culture assays were performed on PDA (-●-) or Vogel’s minimal media supplemented with dextrin (-▼-), cellulose (-●-), glucose (-▲-), glycerol (-◆-), and sucrose (-▅-). RNA was extracted from mycelia after contact in a dual culture assay. The color line identifies carbon sources. Symbols represent average signals, and lanes are SD. *n* = 3.

**Figure 4:**
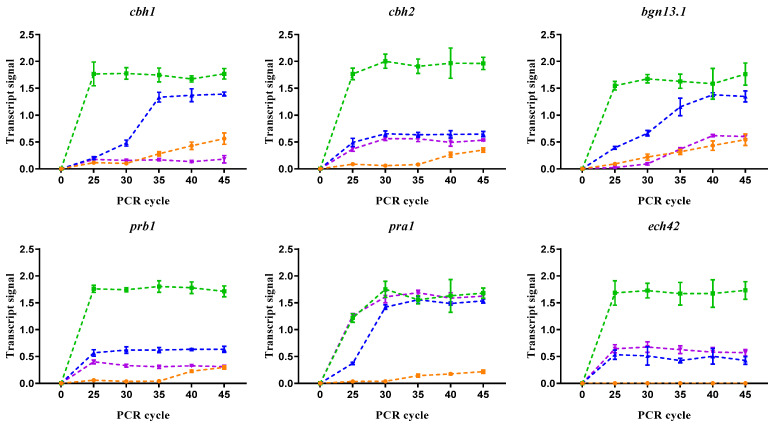
Influence of nitrogen source on the transcript levels of parasitism-related genes during confrontation with NI-1 and D3. Dual culture assays were performed on PDA (-●-) or Vogel’s minimal media supplemented with ammonium nitrate (-▅-), sodium nitrate (-▲-), and L-arginine (-▼-). RNA was extracted from mycelia after contact in a dual culture assay. Symbols represent the average signal, and bars are SD. *n* = 3.

To evaluate the expression of genes encoding hydrolytic enzymes related to parasitism such as cellulases (*cbh1* and *cbh2*), chitinases (*ech42*), endoglucanase (*bgn13.1*), and proteases (*prb1* and *pra1*) during *T. atroviride*–*P. capsici* interaction, primers were designed using the sequences downloaded from the *Trichoderma atroviride* genome available in JGI (Table S1). During *T. atroviride*–*P. capsici* interaction on PDA, the transcripts of GRM analyzed here (*cbh1*, *cbh2*, *ech42*, *bgn13.1*, *prb1*, and *pra1)* were almost undetectable, requiring at least 35 cycles, but were induced during the interaction, except for *ech42* transcript, which was not detected (Figure 3 and Figure 4). The *cbh1*, *cbh2*, and *bgn13.1* encoding glucan hydrolases that degrade the primary cell wall constituents in *P. capsici* were detected at the cycle 40, whereas the *prb1* and *pra1* genes encoding a protease and a trypsin-like protein, respectively, were detected until cycle 45, indicating a very low expression during NI-1–*P. capsici* interaction on PDA plates. PDA is a rich medium containing amino acids and simple and complex sugars [46,47], a condition that could activate CCR and NCR, perhaps repressing the expression of GRM for degradation of *P. capsici* cellular components.

Considering that the very low transcript levels detected on PDA may be affected by a metabolic repression mechanism, Vogel’s minimal medium (VMM) was supplemented with 1% of primary (glucose) or secondary carbon sources such as dextrin, glycerol, sucrose, or cellulose analyzed with ammonium nitrate as a unique nitrogen source. All carbon sources tested stimulated higher transcript levels (Figure 3) than PDA, but dextrin was always better than the other carbon sources analyzed. Transcripts were detected many cycles earlier, and their saturation points on VMM plates indicated an increase in GRM expression levels. The *cbh1* and *cbh2* transcripts were detected starting at cycle 20 when confrontation was performed on VMM supplemented with dextrin, cellulose, and glucose, and their saturation points started at 25 cycles, whereas in glycerol and sucrose, saturation occurred at cycle 30. Similarly, *bgn13.1* transcript levels were higher on VMM supplemented with dextrin and glucose (Figure 3).

Protease expression was also influenced by carbon source; *prb1* and *pra1* transcript levels were detected at earlier cycles, reaching saturation at 25 and 30 cycles, respectively, in media supplemented with glucose, sucrose, and dextrin. As indicated previously, when *P. capsici* and NI-1 confrontation was carried out on the PDA medium, *ech42* transcripts were not detected, whereas on VMM supplemented with dextrin and glucose, high expression levels were observed. Although glycerol does not interfere with the induction of cellobiohydrolase when *Trichoderma reesei* grows in cellulose [48], all GRM transcripts were expressed at lower levels during the interaction between *T. atroviride* and *P. capsici* on VMM with glycerol as a carbon source. Interestingly, glucose, considered the primary carbon source, was documented to repress genes encoding lytic enzymes, but its activity had no impact on the induction of GRM expression in this assay. These results suggest that dextrin and glucose favor a high expression of genes encoding hydrolytic enzymes during *T. atroviride* parasitism.

### 3.4. Primary Nitrogen Sources Promoted Mycoparasitic-Related Gene Expression

Considering that the preferent nitrogen sources trigger repression to metabolize secondary sources, two secondary nitrogen sources (sodium nitrate and L-Arginine) were used and compared with the preferential nitrogen source (ammonium) (Figure 4). Notably, the preferred nitrogen source favored the transcript levels detected for all genes; the relative expression was consistently higher, reaching early saturation when the nitrogen source was ammonium nitrate. Only the *pra1* gene reached high transcript levels independent of the nitrogen source. These results suggest that the mechanisms of nitrogen metabolism repression do not operate on GRM genes during a parasitic interaction between *T. atroviride* and *P. capsici*.

### 3.5. Trichoderma Atroviride Can Distinguish Its Prey

**Figure 5:**
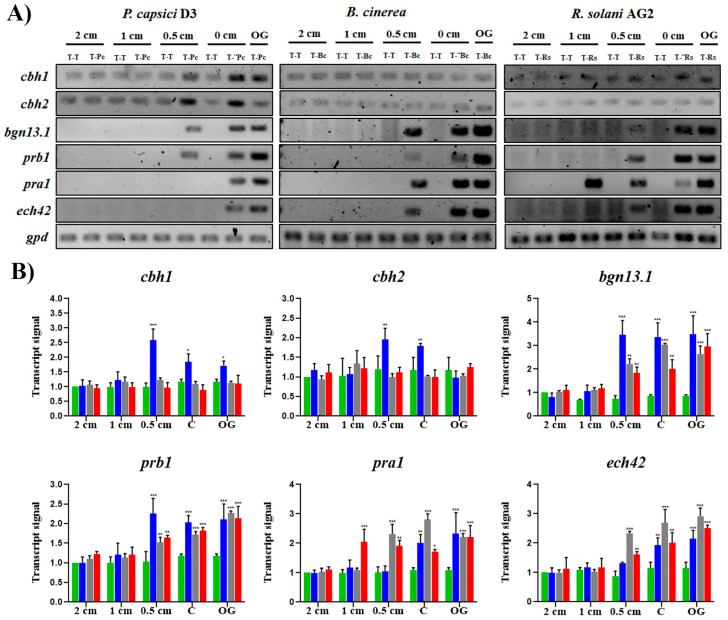
The GRM expression during the confrontation of NI-1 against three different phytopathogens. (**A**) Gene expression profile during mycoparasitic interaction in dual cultures: before contact (2, 1, 0.5 cm), physical contact (0 cm), and overgrowth (OG). Interactions of *T. atroviride* against *P. capsici* (T-Pc), *B. cinerea* (T-Bc), and *R. solani* (T-Rs) were analyzed using as a control *Trichoderma*–*Trichoderma* (T-T). (**B**) Relative gene expression. The signal area detected before contact (2, 1, 0.5 cm), physical contact (C), and overgrowth (OG) in (A) was quantified, and gene expression was graphed for T-Pc (blue bars), T-Bc (gray bars), and T-Rs (red bars) relative to the control T-T, which was adjusted to the unit (green). The transcript levels of *gpd* were used to normalize the template amounts for each signal. Two-way ANOVA and multiple comparisons were performed using Dunnett’s test. Asterisks above the bar represent significant differences with respect to control relative expression *p* < 0.05 (*), *p* < 0.001 (**), and *p* < 0.0001 (***). Bars represent average transcript levels, and the lines indicate SD. *n* = 3.

It has been documented that *Trichoderma* detects its host at a distance by diffusible factors [18], redirecting its growth to reach the host. To analyze the expression of GRM during *T. atroviride*–phytopathogen interaction, a confrontation assay was carried out, analyzing transcript levels before contact, contact, and overgrowth using minimal medium supplemented with dextrin and ammonium (Figure 5 and Figure S3). 

All GRMs analyzed were generally detected before contact with the host, suggesting that diffusible factors regulate their expression. Interestingly, the specific transcript levels varied depending on the host, suggesting that different molecular mechanisms to detect the host are involved in *T. atroviride*. The background of *cbh1* and *cbh2* gene expression was detected throughout all stages of confrontation with all phytopathogens, which may be provoked by the cellophane used. The *cbh1* and *cbh2* genes were specifically induced during the confrontation against *P. capsici* at 0.5 cm before contact and physical contact. An increase in transcript levels was observed, which decreased during overgrowth (Figure 5A,B), whereas their expression was unresponsive during interaction with either *R. solani* or *B. cinerea*.

The *bgn13.1* and *prb1* transcripts were detected during all confrontation assays of *T. atroviride* NI-1–phytopathogens, indicating that the expression of both genes is indistinctly induced by the presence of a host when confronted with a fungus or an oomycete. Transcripts of both genes were detected at 0.5 cm before contact and remained high during the contact and overgrowth but were significantly higher during the confrontation with *P. capsici* concerning *B. cinerea* and *R. solani*, which had similar relative expression (Figure 5B). 

Also, *pra1* and *ech42* expression were induced by the host presence; however, during the interaction of NI-1 and *P. capsici*, both genes were detected until they were in physical contact and maintained their high level during overgrowth (Figure 5A,B). During interaction with *B. cinerea* and *R. solani*, both genes were induced at 0.5 cm before contact, but the *pra1* gene was induced at 1 cm before contact with *R. solani*. These results suggest that *Trichoderma* can distinguish between a fungus and an oomycete, and that *Trichoderma* detects different signals from a host, both diffusible factors, and by contact with some surface structural components.

The impact of nutrients on *T. atroviride* parasitism was more evident on *ech42*; it was not detected during the NI-1–*P. capsici* interaction on PDA medium (Figure 3 and Figure 4); however, when the interaction was carried out in VMM, it was clearly detected, including when they were in direct contact and overgrowth (Figure 5A). Together, the patterns of GRM expression analyzed when NI-1 confronted *B. cinerea* and *R. solani* were similar, but different during interaction with *P. capsici*, indicating different molecular mechanisms that *T. atroviride* has when detecting its host.

## 4. Discussion

Although *Trichoderma* parasitism has largely been studied to improve plant protection, and several species have been sequenced with data available online, the molecular mechanisms of how *Trichoderma* senses and transduces signals related to the hosts, which chemical signals of the hosts trigger parasitism of *Trichoderma*, and how genes related to parasitism are regulated remain to be elucidated [1].

Multiple factors influence mycotrophy, including the host–pathogen, abiotic stresses, and nutrient availability [17]. Based on our results, the aggressive behavior of the NI-1 strain under the conditions evaluated here is due to the carbon and nitrogen sources and phytopathogens. The carbon source significantly impacted GRM expression during the *T. atroviride*–*P. capsici* interaction. Although the PDA medium limited the expression of the GRM, *T. atroviride* could detect and respond to the host. Notably, responsiveness was stronger when the interaction was carried out on MMV supplemented with glucose and dextrin; the latter is a starch byproduct, and both are the primary carbon sources available in the PDA medium. Although glycerol and glucose are generally considered neutral and repressive carbon sources, respectively, they are known not to influence the expression and secretion of hydrolytic enzymes in *Trichoderma* [48]. Our results indicate that the *T. atroviride* NI-1 strain can express the analyzed GRM when interacting with a potential host despite these carbon sources. Similar results were observed when *T. hamatum* expressed *prb1* and *ech42* while interacting with *Sclerotinia sclerotium* in the presence of glycerol [10] or *T. asperellum* expressing a glucanase in the presence of glucose [49]. Considering the above, we suggest that *T. atroviride* NI-1 possesses a mechanism that prevents the CCR, ensuring the production of an enzymatic arsenal that inhibits the growth of a competitor. In addition, unlike the previous approaches used to analyze *Trichoderma* parasitism in a low-carbon-available condition [10] that triggers the process of autophagy and cell death in fungi [50], our results suggest that synthetic mediums’ preferent carbon and nitrogen sources improve responsiveness in *Trichoderma*. 

The genes encoding cell wall degrading enzymes are induced by their specific substrates, such as glucan, chitin, cellulose, protein, and fungal cell walls [7,17,51]. Preferent nitrogen and carbon sources repress gene expression, and derepression by starvation is necessary to induce their expression in vitro. For example, the expression of *prb1* was induced when *T. atroviride* IMI206040 grew in the presence of chitin, autoclaved mycelia, or *R. solani* dried cell walls, but not in the presence of glucose [52]. During interactions between *T. atroviride*–phytopathogens in the presence of preferent carbon and nitrogen sources, expression of the GRM was induced, indicating that *Trichoderma* prefers the parasitic lifestyle. Furthermore, these findings suggest that CCR or NCR, the main molecular mechanism devoted to sense the preferent nutrient to optimize resources, could be influenced by signals attributable to the living host. Our results reinforce the hypothesis that mycoparasitism is an ancestral trait in the *Trichoderma* lineage [26] and a dominant characteristic of *T. atroviride*.

It was observed that the expression of GRM is enhanced when both organisms compete for the consumption of preferent substrates. The oligosaccharides such as dextrins used in this research are considered ubiquitously used by *Trichoderma*, *Phytophthora*, and other fungi [53]. Our results showed that dextrin from starch is a carbon source that does not repress the expression of the genes analyzed here without suppressing their parasitic performance. In that sense, starch is a carbon source that induces the expression of glucanase *tag83* in *T. asperellum* [49]. Therefore, another hypothesis is that *Trichoderma* employs parasitic strategies to eliminate competitors, ensuring that the substrate is available. To explain the lower responsivity of *Trichoderma* on PDA means that preferent nitrogen source ammonium is also key to attacking its prey. PDA has high levels of glucose and other carbohydrates present in potatoes, but nitrogen is available mainly in the form of amino acids and proteins.

The availability of carbon or nitrogen triggers signaling pathways that influence the development of *Trichoderma*. For example, nutritional stress can activate the TOR pathway by negative regulators, and the absence of these regulators decreases the mycoparasitic activity of *T. atroviride* as well as vegetative development [54]. In that sense, when we elevated the concentrations of carbon and nitrogen to reduce cellular stress, GRM expression increased, suggesting another mechanism that deregulates the activation of signaling pathways and allows the assimilation of alternative carbon and nitrogen sources. 

The GRM expressions during interaction with NI-1–*R. solani* and NI-1–*B. cinerea* were similar, but they were different during interaction with NI-1–*P. capsica* under the same conditions, suggesting that *T. atroviride* can differentiate between a true fungal and an oomycete, linked to specific combat strategies. *Trichoderma* detected phytopathogens before contact, suggesting that specific diffusible factors associated with the prey were detected. It was documented that a small organic molecule is capable of passing across a cellophane membrane, activating the mycoparasitic response in *T. atroviride* [18]. Another explanation could be related to differences in cell wall composition [55] or proteins secreted [56] specific to *P. capsici* in relation to ascomycetes or basidiomycetes. For example, elicitins that are specific to oomycetes and trigger a defense response in tobacco plants [57] can act as MAMPs that also trigger a molecular defense response in the NI-1 strain, which includes the expression of GRM.

The *cbh1* and *cbh2* genes encode two cellobiohydrolases, which are of biotechnological interest for the degradation of cellulose in *T. reesei* [58]. However, few reports have suggested that the intervention of this group of genes is related to mycoparasitism [59]. Based on our results, we suggest that *cbh1* and *cbh2* play a role in the parasitism of *P. capsici* at the onset of the interaction but not with *R. solani* or *B. cinerea*. In this sense, the *P. capsici* cell wall is mainly composed of β-glucans, cellulose, and proteins [60], and small amounts of chitin in their cell wall are associated with vegetative growth, asexual reproduction, and pathogenicity [61]. However, modification of cell wall composition is a process described by many phytopathogens; therefore, *P. capsici* could modify their cell wall during parasitic interactions with *T. atroviride* NI-1 and influence the *cbh1* and *cbh2* gene expression to protect their cell integrity [62]. Similar results were observed during the interaction of *T. reesei* with the oomycete Pythium ultimum in which the *cbh1* transcript was upregulated when in the presence of the phytopathogen [63].

The *Bgn13.1* gene encodes an endo-β-1,3 glucanase that is involved in extracellular digestion of the cell wall components of the host fungus. In this case, we observed the influence of phytopathogen on the expression of *bgn13.1*, as was reported in *T. harzianum*, *T. asperellum*, and *T. atroviride* [1]. Expression of *pra1* does not seem specific for any phytopathogen, as has been reported for other *Trichoderma* strains. In this sense, *pra1* is associated with extracellular proteolytic activities, as we observed in our results because expression occurs before contact, similar to the interaction between *T. atroviride* IMI206040 and the phytopathogens evaluated here [10]. However, the expression levels could vary depending on the host and stage of interaction, suggesting that the expression of *pra1* could be influenced by specific proteins or metabolites from the host. *Ech42* is known to be induced by fungal hosts, as well as NI-1, when interacting with *R. solani* and *B. cinerea*. However, few reports about the role of this gene during the colonization of oomycetes have been carried out. The NI-1 strain expressed this gene only during the contact and colonization processes, suggesting that the induction of *ech42* of NI-1 occurs to counter the defense mechanisms from the oomycete. A similar response was observed on the tripartite interaction system plant–*Trichoderma*–*Phytophthora nicotianae* in which *ech42* of *T. asperellum* and *T. atroviride* were upregulated when the systems confronted the phytopathogen [64]. 

In general, the nutritional status of *T. atroviride* NI-1 during the antagonistic interaction against a competitor is essential to performing a good parasitism process, in which the expression of genes related to mycoparasitism is involved, and the host to be colonized can be specifically detected. In that sense, the induction of GRM tends to be upregulated in the presence of the fungal host independently of the carbon and nitrogen source in the media. 

Considering that CCR and NCR are economizing mechanisms for saving energetic resources on fungi, the transcription of GRM for assimilation of the host’s cell wall carbohydrates and proteins is needed to counter the presence of competitors despite repressive nutriment sources. Consequently, the presence of the host may block the economizing mechanisms to transform *T. atroviride* NI-1 into a successful competitor. Based on the expression pattern of genes analyzed before contact, *Trichoderma* can distinguish between an oomycete and a true fungus, indicating the presence of different molecular mechanisms to detect and attack its prey. Therefore, *T. atroviride* NI-1 hydrolytic enzymes involved in parasitism during confrontation with *R. solani*, *B. cinerea*, and *P. capsici* have a different expression pattern, which might be influenced by specific host metabolic and/or protein-secreting profiling.

## 5. Conclusions

Environmental factors influence the genetic repertoire expressed by an organism. A dominant regulation of preferent carbon and nitrogen sources on gene expression encoding enzymes to degrade and potentially assimilate the main structural components of the plant pathogens was documented. Consequently, coculture assays to analyze the *Trichoderma* parasitism were performed on a culture medium limited in carbon and nitrogen sources, implying that the presence of the host elicits stimuli that upregulate the genes involved in consuming structural biomolecules from prey. In this case, parasitism and aggressive behavior of *Trichoderma* must also be considered as possible mechanisms that ensure their survival. Otherwise, confronting assays between *T. atroviride* NI-1 and *P. capsici*, *R. solani,* or *B. cinerea,* respectively, showed that *Trichoderma* can distinguish between oomycetes and fungal hosts. The high parasitic responsiveness exhibited by *Trichoderma* on preferent carbon and nitrogen sources supports experimental evidence that parasitism is an ancestral lifestyle shared by many strains.

## Figures and Tables

**Table 1:** GenBank accession numbers for reference sequences used in phylogenetic analysis.

Species	Strain	Source	Country	ITS	Tef-1α	Reference
*T. pubescens*	GJS 01-207	Bark	Cameroon	EU856280	EU856304	[25]
*T. atroviride*	NI-1	Soil	Mexico	PQ162586	PQ335171	This work
*T. atroviride*	IMI 206040	Soil	Sweden	AF278795	300828 ^1^	[26]
*T. atroviride*	P1	Soil	UK	OQ360634	EF581849	[27]
*T. atroviride*	DAOM 231423	Soil	Mexico	EU280111	EU280002	[28]
*T. asperelloides*	GJS 04-217	*Theobroma cacao*	Peru	DQ381957	DQ381958	[29]
*T. asperelloides*	GJS 99-6	Wood	USA	DQ315464	GU198240	[29]
*T. asperellum*	TUB-F755	Soil	Mexico	AY857217	AY857273	[30]
*T. asperellum*	CBS 433.97	Sclerotia	USA	AY380912	AY376058	[31]
*T. asperellum*	GJS 90-7	Soil	Vietnam	GU198317	EU338333	[29]
*T. yunnanense*	CBS 121219	Soil	China	GU198302	GU198243	[29]
*T. gamsii*	GJS 05-111	*Ricinus comunis*	Italy	DQ841730	DQ841722	[32]
*T. koningiopsis*	LESF212	*Cyphomyrmex wheeleri* nest	USA	HQ608031	KT278985	[33]
*T. koningiopsis*	GJS 93-20	Branch	Cuba	DQ313140	DQ284966	[32]
*T. viride*	GJS 05-104	Peat	Italy	DQ841741	DQ841727	[34]
*T. trixiae*	CBS 189.79	Wood	Italy	KJ482546	KJ482552	[35]
*T. ovalisporum*	DAOM 229859	Soil	Panama	EU280118	EU280003	[28]
*T. erinaceum*	GJS 02-103	Wood	Sri Lanka	KR873100	KR873098	[36]
*T. tomentosum*	CBS 349.93	ND ^2^	Canada	MH862417	AF401024	[37]
*T. velutinum*	DAOM 230014	Soil	Nepal	DQ083010	AY937446	[38]
*T. harzianum*	DAOM 231646	Soil	South Africa	AY605723	AY605766	[39]
*T. harzianum*	DAOM 229907	Soil	USA	AY605734	AY605777	[39]
*T. reesei*	GJS 97-178	*Theobroma* sp.	French Guiana	AJ004964	GQ354348	[40]
*T. saturnisporum*	CBS 886.72	*Triticum* sp.	South Africa	X93974	AY937414	[38]
*T. longibrachiatum*	DAOM 166989	Soil	Canada	EU330961	EU338335	[41]

^1^ Accession number is from protein ID and sequence obtained from the JGI database. (https://mycocosm.jgi.doe.gov/cgi-bin/dispGeneModel?db=Triat2&tid=300828 accessed on 8 August 2024). ^2^ ND: not determined by the author.

## Data Availability

The original contributions presented in the study are included in the article/Supplementary Materials, further inquiries can be directed to the corresponding author.

## Supplementary Materials

The following supporting information can be downloaded at: https://www.mdpi.com/article/10.3390/jof10100671/s1. The supplementary material comprises Figure S1: Influence of NI-1 Volatile Organic compounds (VOCs) over *P. capsici* D3. Figure S2: Colonial morphology of NI-1. Figure S3: Plate confrontations between *T. atroviride* NI-1 against phytopathogens. Table S1: Primers sequences used to analyze gene expression by semi quantitative RT-PCR. References [45,65,66] are cited in the supplementary materials.
