# Supplementary material for: Carbon and Nitrogen Sources Influence Parasitic Responsiveness in Trichoderma atroviride NI-1
Source: J Fungi (Basel). 2024 Sep 26;10(10):671. doi: 10.3390/jof10100671 (PMC11508198; doi:10.3390/jof10100671)
Supplement: Supplementary file 1 [file jof-10-00671-s001.zip › jof-3206275-supplementary.pdf]

Supplementary material

Table S1. Primers sequences used to analyze gene expression by semi quantitative RT-PCR.

Gene[1]	Forward (5′-3′)	Reverse (5′-3′)	Amplicon size (pb)	CT[2]	GenBank accession number	Reference
prb1 (166201)	CATCAGCAATTCGGCGGGC	CCATTACCAGCGGCGACAATG	327	23	XM024901871	This study
pra1 (25266)	CCAGGTTGGTGTTTCAAGCATC	GAGCAGGAGTCCTTGCCACC	326	23	XM024903604	This study
cbh1 (46985)	GGGCAACCACACTTTCTATGG	GAAGCGGTATCGTTGGTTGGG	339	22	XM024906693	This study
cbh2 (84972)	GGAGGCAACTACGCTGGTC	CAGCCAAGCCATCCTGCGTG	334	23	XM024910837	This study
bgn13.1 (167916)	GCATCGTCGGTGAGGGTTG	CACCTCCCTTTCCTGCAATAG	401	23	XM024901914	This study
ech42 (72752)	CTATGCTGCGCAATACGCTCC	AACGATCTTGCTTGCGGGAAC	264	23	XM024910215	This study
gpd (56409)	CACCACCAACGGCCTGGCTCC	GGACATGCCGGTGATCTTGCCG	220	27	XM024907810	[45]
ITS	TCCGTAGGTGAACCTGCGG	TCCTCCGCTTATTGATATGC	≈550	ND	ND	[65]
Tef-1α	CATCGAGAAGTTCGAGAAGG	TACTTGAAGGAACCCTTACC	≈1100	ND	ND	[66]

[1] Associated to GenBank identifier (Access on November 27th 2023)

[2] CTs based on number of cycles required to reach linear amplification.

A)

	% Inhibition	Standard deviation
VOCs	69.17	± 4.86
Diffusible metabolites	100.00	± 1.36

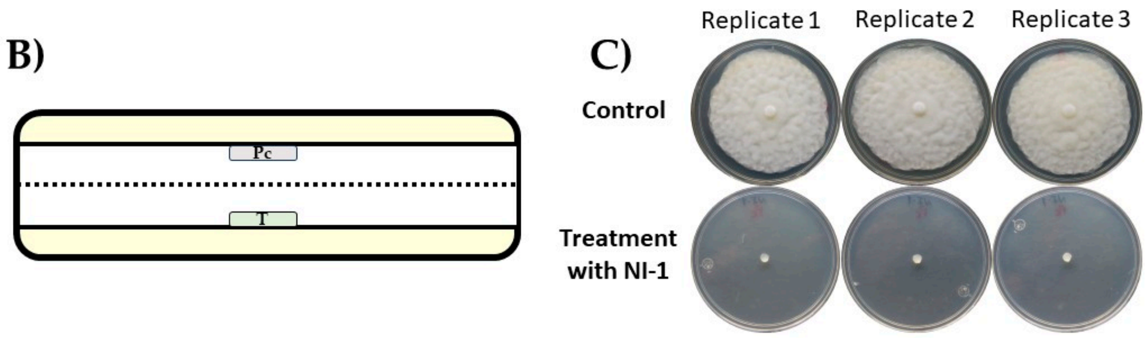

Figure S1. Antagonistic activity of NI-1 against *P. capsici* D3. A) Growth inhibition of *P. Capsici* D3 by VOCs and diffusible metabolites. B) Schematic overview of two opposing petri dishes and sealed together. T plug of 5 mm of diameter of *T. atroviride* NI-1. Pc plug of 5 mm of *P. capsici* D3. VOCs from NI-1 impact directly on *P. capsici* D3. C) Diffusible metabolites assays. The Mycelium of *T. atroviride* NI-1 grew over a cellophane layer, then removed, and a plug of *P. capsici* D3 mycelium was placed over the same petri dish. As a control, fresh PDA plates were inoculated with P. capsici plugs.

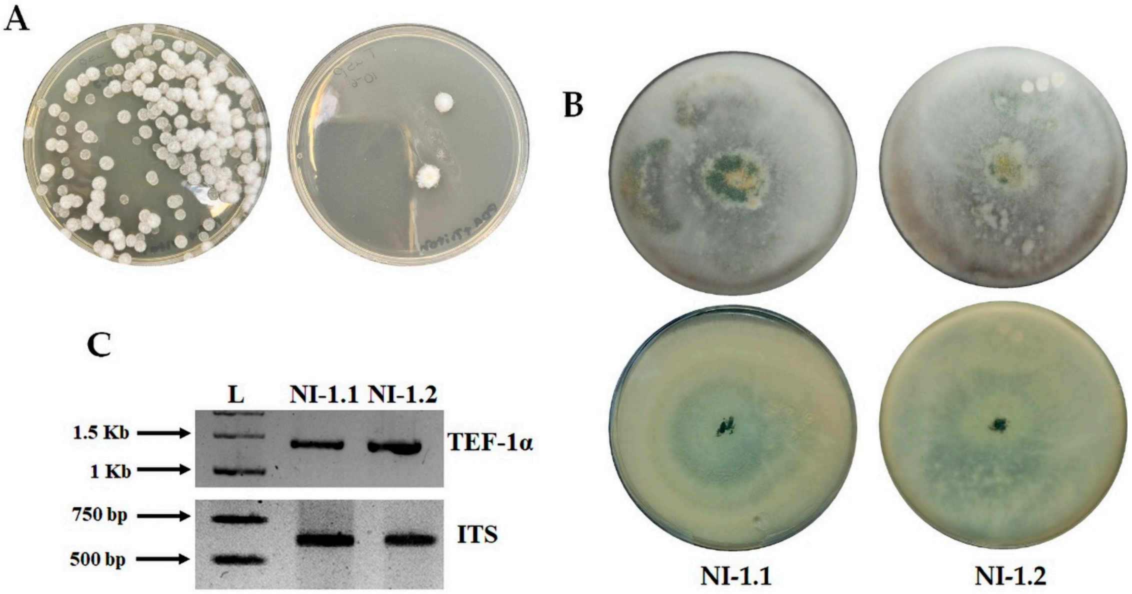

Figure S2. Colonial morphology of the *Trichoderma* isolate NI-1 and the amplification of the indicated molecular markers. **A**, Pure colonies obtained from monosporic cultures at dilution 10^{-7} and 10^{-8} respectively. in PDA medium supplemented with chloramphenicol and Triton X-100. Colonies maintained at 28°C under constant light for 3 days. **B**, Pure cultures of NI-1.1 and NI-1.2 growth on PDA medium at 28°C under constant light. The colonies were randomly selected and propagated on PDA plates. Up front of plates. Down back of plates. **C**, Amplicons for TEF-1α and ITS molecular markers using total DNA from mycelia grew in PDB for 48 h at 27°C, shaken at 150 rpm. The size of the amplicons was ≈ 1300 and ≈ 650 pb respectively. L lane correspond to DNA ladder of 1 Kb

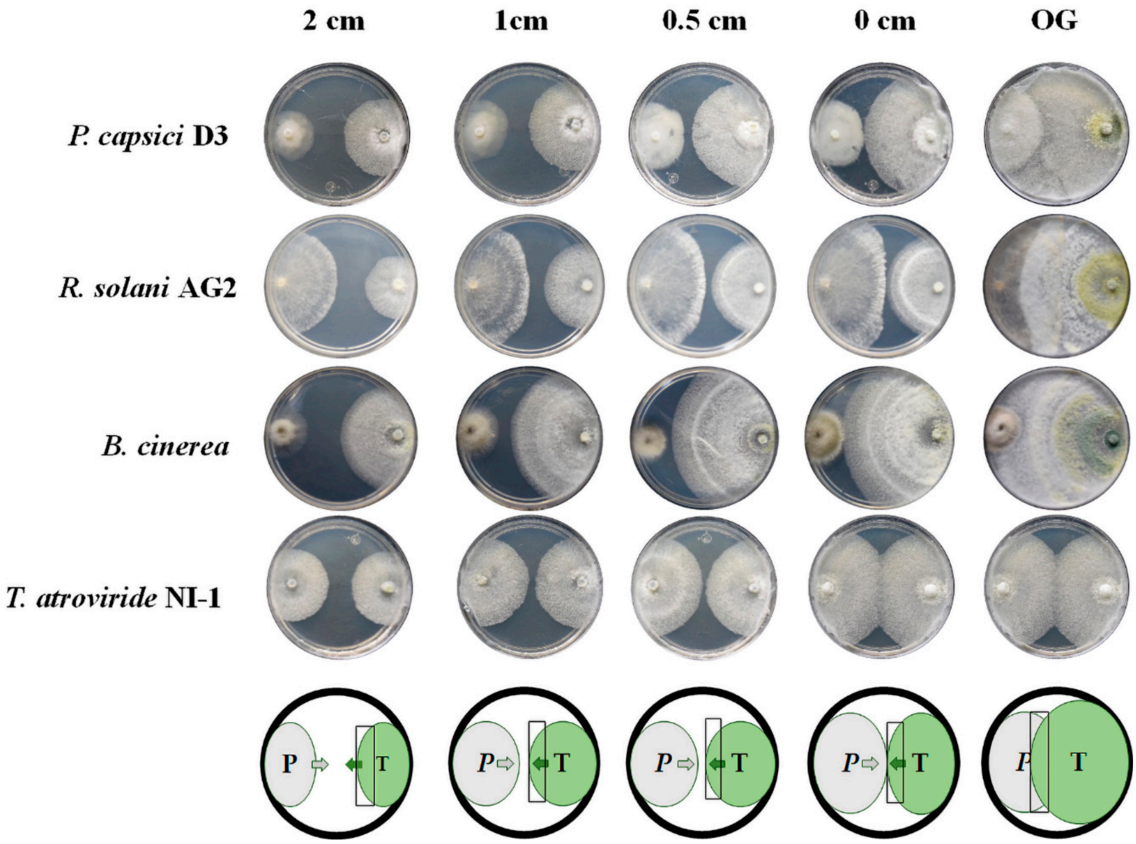

Figure S3. Confrontations in dual cultures between *T. atroviride* NI-1 against phytopathogens. Interactions were carried out on Vogel's minimal media with dextrins from potato starch as a carbon source. Mycoparasitism behavior monitored previous contact (2 cm, 1 cm and 0.5 cm), contact (0 cm) and overgrowth (OG). Squares indicate where mycelium for RNA isolation was obtained. **T**, *Trichoderma atroviride* NI-1. **P**, phytopathogen as a potential host to be colonized.
